# Introduction to ‘Cognitive artificial intelligence’

**DOI:** 10.1098/rsta.2022.0051

**Published:** 2023-07-24

**Authors:** Alan Bundy, Nick Chater, Stephen Muggleton

**Affiliations:** ^1^ The University of Edinburgh, Edinburgh, Edinburgh, UK; ^2^ Warwick Business School, University of Warwick, Coventry, West Midlands, UK; ^3^ Computational Bioinformatics Laboratory, Imperial College London, UK

## 1. Introduction

There is an increasing excitement concerning the potential of artificial intelligence to both transform human society and to understand cognition in humans and other animals. This meeting addressed the leading edge of research intersection of artificial intelligence and cognitive science, an area we are calling *cognitive artificial intelligence*. Topics covered include:

— Improving the interaction between humans and machines, including how machines can explain their reasoning to humans, might be more socially aware and understand a human's beliefs and intentions.
— Contrasting how machines and humans learn, and showing how machines might emulate humans in learning from only a few examples and how machines can aid the teaching of humans.
— How reasoning and learning interact, including how failures of reasoning trigger the evolution of models of the environment.
— The contrast between symbolic and subsymbolic reasoning, especially the role of large language models, such as ChatGPT, in generating natural language and serving as a model of human cognition.

This special issue is the proceedings of a Royal Society Hooke Meeting on Cognitive artificial intelligence. The meeting was proposed by the EPSRC Network+ on *Human-Like Computing* (HLC). According to the influential US funding agency DARPA (originator of the Internet and Self-Driving Cars), this new area represents the third wave of artificial intelligence (3AI, 2020s–2030s), and is being actively investigated in the USA, Europe and China. The HLC Network was one of the first internationally to initiate and support research specifically in this area. Starting activities in 2018, the Network represents around 60 leading UK groups of artificial intelligence and cognitive scientists involved in the development of the inter-disciplinary area of HLC. The research of network groups aims to address key unsolved problems at the interface between Psychology and Computer Science.

## 2. Objectives

The key fields brought together at this meeting are artificial intelligence and cognitive science. This meeting helped forge better understanding and interactions in a joint area which we refer to as cognitive artificial intelligence. Artificial intelligence and machine learning are becoming centrally relevant to a variety of sciences in supporting the construction of complex models from data. Furthermore, within society at large artificial intelligence is viewed as having both immense potential for enabling human societies, while simultaneously presenting dangers for weakening the social fabric. It is clear that advances in understanding of how to build automated learning systems which are compatible with human understanding, planning and reasoning has immense potential for beneficial effects in many areas. However, interactions of cognitive scientists with leading edge artificial intelligence research requires many advances and new experimental work in psychology to further understand the cognitive and social constraints of human beings when interacting with machines. The meeting allowed presentations on the latest results from leading laboratories in this area, as well as encouraging discussion on key topics for joint research between artificial intelligence and cognitive science groups.

## 3. Significance

Both artificial intelligence and cognitive science have a variety of large-scale annual conferences. However, researchers within each of these areas typically have limited understanding of advances in each other's fields. The meeting helped bring together leading scientists from artificial intelligence and cognitive science to inform each other of key open questions that joint work could help address.

## 4. Social implications

In recent years, there has been increasing public concern about the application of artificial intelligence. Such concerns were documented within the House of Commons and Lords Select Committee Reports and the Royal Society Report on Machine Learning. Key issues raised included those of the need for (a) transparent decision making, (b) accountability and its related legal implications, (c) safety of automated systems in control tasks and (d) the threat to jobs. Research in the new area of cognitive artificial intelligence will aim to advance fundamental understanding for the key artificial intelligence technologies being developed. This meeting has the potential to promote understanding of the advances required to go beyond the development of simple black-box decision makers to allow the development of systems which take account of our understanding human modes of perception and social interaction. Such advances have potential for wide-ranging social benefit.

## 5. Points of view

Since both artificial intelligence and cognitive science are both well-established fields, there is considerable diversity of viewpoints within each field. Within artificial intelligence, this tends to be related to the choice of representation used for representing knowledge, while in Cognitive Science, there are a wide variety of differences in methodology and experimental techniques. The meeting was devised to include representative from these diverse communities and viewpoints, with the aim of encouraging wide-ranging discussion.

## 6. Overview of the contributions

This issue of Cognitive artificial intelligence consists of 11 substantive papers, drawn roughly equally from the artificial intelligence and cognitive science communities, but each drawing on and having relevance to both.

We begin with Muggleton's [1] paper ‘Hypothesizing an algorithm from one example: the role of specificity’ which argues that while the dominant methods of Statistical Machine Learning achieve high accuracy, they require large numbers of examples to do so. By contrast, humans typically learn new concepts from as few as one example. However, the high data efficiency of human learning cannot be explained by existing standard formal frameworks for machine learning. Muggleton shows that this disparity can be resolved by introducing a revised Bayesian framework for expected error, and shows that highly specific concepts, as typified by computer algorithms, can be learned within this theoretical framework with high accuracy from a single example, by using a preference for specificity combined with minimality. Experiments with Muggleton's implementation of this approach, called DeepLog, indicate that such an approach can be used in practice to efficiently construct relatively complex logic programs from a single randomly selected example.

Wahlster's [2] paper ‘Understanding computational dialogue understanding’ first explains why human-like dialogue understanding is so difficult for AI. It discusses various methods for testing the understanding capabilities of dialogue systems. It reviews the development of dialogue systems over five decades, focusing on the transition from closed-domain to open-domain systems and their extension to multimodal, multiparty and multilingual dialogues. From being somewhat of a niche topic in artificial intelligence research for the first 40 years, it has made newspaper headlines in recent years and is now being discussed by political leaders at events such as the World Economic Forum in Davos. It asks whether large language models are super-parrots or a milestone towards human-like dialogue understanding and how they relate to what we know about language processing in the human brain. Using ChatGPT as an example, it presents some limitations of this approach to dialogue systems. Finally, it presents some lessons learnt from 40 years of research in this field about system architecture principles: symmetric multimodality, no presentation without representation and anticipation feedback loops. It concludes with a discussion of grand challenges such as satisfying conversational maxims and the European Language Equality Act through massive digital multilinguality—perhaps enabled by interactive machine learning with human trainers.

The next article, ‘Symbols and grounding in large language models' by Pavlick [3], considers the practical and theoretical significance of large language models, which have in the last few years been shown to carry out a large number of open-ended natural language tasks with often close to human levels of performance on some measures. These models consist of very large deep neural networks trained on a substantial fraction of the entire contents of the World Wide Web. Within the cognitive science community, many have argued that models trained purely on a large amount of language data, however impressive their performance, are inevitably restricted in their relevance to human cognition. Pavlick takes up two specific charges against the cognitive relevance of such models and argues for a verdict of ‘not proven' in both cases. The first issue she addresses is that large language models are not endowed with structured symbolic representations, which are widely presumed to underpin perception, thought and language in humans. But she notes, drawing on her research, that the internal representations learned by large language models may actually have a distinctly symbolic character, and that the nature of such representations can only be determined by sophisticated analysis of how large language models work. The second issue is that large language models are sometimes presumed not to be ‘grounded' through the perceptuo-motor interaction with the world (although links between large language models and models of visual processing are relatively advanced). But in any case, Pavlick argues that modern philosophy of language assumes that the grounding of linguistic symbols is a collective achievement, at the level of the entire language community, rather than operating individual by individual. Thus, the lack of direct grounding may raise no special difficulties for large language models, even if these are not integrated with modules for perception and action. They may thus inherit grounded symbols from the human language on which they are trained.

We next move from language to mathematical and scientific cognition. In ‘DreamCoder: growing generalizable, interpretable knowledge with wake-sleep Bayesian program learning', Ellis *et al*. [4] introduce a system that learns to solve a wide range of representationally challenging problems by learning to write programs. Combining symbolic and neural network methods, it learns programming languages for capturing concepts relevant to the target domain, and uses neural networks to guide the search process to create appropriate programs using these languages. They apply a ‘wake-sleep' algorithm, which interleaves the extension of the programming language with new symbolic abstractions and training the neural network on imagined and past problems. Dreamcoder can be applied successfully to a wide range of problems, from drawing pictures and building scenes, to rediscovering the fundamentals of functional programming, vector algebra and classical physics, including Newton's and Coulomb's Laws. Learning operates by successively creating new abstractions from previous abstractions, creating rich systems of representation which transfer effectively across task domains.

Goodman & Poesia [5] continue the theme of how machines can learn to engage in rich, structured representation and reasoning, now focusing on mathematics, in their paper ‘Peano: learning formal mathematical reasoning'. They note that while mathematics is created slowly, involving a huge collective intellectual effort over many centuries, it can relatively rapidly be taught afresh to each generation of students, who can learn to apply it successfully also from a very limited set of training examples. Goodman & Poesia argue that fundamental to mathematical discovery and learning is the ability to create and reason over representations at ever-increasing levels of abstraction. The computational model, Peano, is a theorem proving environment which has the power to represent a wide range of aspects of mathematics. They show that attempting to learn mathematical regularities using traditional reinforcement learning methods is unsuccessful; but adding the ability to learn reusable abstractions (which they call ‘tactics') from past problem-solving attempts, allows the agent to make cumulative progress. The way in which these abstractions are generated sheds light on the ‘natural' order in which such abstractions should most helpfully be presented human learners—and this order agrees to a substantial degree with the order in which ideas are introduced in learning curricula for human learners, such as that used in the Kahn Academy. Their work raises the possibility that deeper understanding of learning mathematics using automated methods may shed substantial might both on the process by which humans learn mathematical concepts, and the optimal design of mathematical curricula.

Bundy & Li's [6] paper ‘Representational change is integral to reasoning' proposes a mechanism by which language evolves in response to reasoning failures. For instance, concepts may be split (mother into birth mother and step mother) or merged (Morning Star and Evening Star into Venus) when current theories either predict things observed to be false or fail to predict things observed to be true. They start by illustrating that concept evolution occurs even in mathematics. An examination of Imre Lakatos's classic rational reconstruction of the history of Euler's Theorem (*V + F − E = 2*) about polyhedra shows that the initial concept of *polyhedron* was not fully defined and that potential counter-examples can be included or excluded depending on how this initial definition is refined. They then discuss their ABC system that evolves logical theories by a combination of abduction, belief revision and conceptual change which reconcile an initial theory's predictions with conflicting observations of the environment, leading to a revised theory.

The next article, ‘Argument and explanation' by Hahn & Tesic [7] considers the relationship between the concepts of argumentation and explanation in the context of philosophy of science and common-sense reasoning. While the philosopher Carl Hempel saw scientific explanation as a type of argument, Hahn and Tesic stress that arguments typically play a role in dialogue, in trying to convince others (and perhaps also oneself) of the truth or rightness of some contested matter. Here, arguments are in the service of the broader goal of persuasion, and factors beyond the argument itself (such as who was its source) are crucial. But explanations can often apply when there is no issue of doubt about the point to be explained: thus, facts ranging from, say, the blueness of the sky or that a piece of kitchen cheese has been nibbled, may not be in doubt, but still may stand in need of explanation. A crucial issue here is what makes an explanation satisfying—what distinguishes chains of reasoning to a particular conclusion that provides a sense of insight and understanding. Hahn & Tesic provide a review of the state-of-the-art in psychological and atificial intelligence approaches to both argument and explanation, and point the way for future research.

Gweon *et al*. [8] in their paper ‘Beyond imitation: machines that understand and are understood by humans' focus on a particular, and especially fundamental, aspect of explanation: the human ability to infer and reason about the mental states of others from observing their behaviour. Such inferences may be crucial when attempting to learn from another person; and equally is crucial from the point of view of the teacher, attempting to infer what the learner already knows and which actions or words will best help them learn successfully. This type of social intelligence develops early in humans, but seems difficult to replicate in machines: artificial intelligence systems currently have a limited ability to understand, or be understood by, humans with which they interact. Gweon *et al.* argue that a central goal of AI should be the creation of genuinely socially intelligent machines, that model and consider the minds of people they interact with, rather than more superficial social niceties, such as mimicking human facial expressions, gestures, or patterns of speech. They survey work on the development of human social intelligence, and human–machine interaction, and argue that this could provide crucial clues for how to create a new generation of machines that can engage in rich social interactions with people. Indeed, they argue that integrating cognitive science and artificial intelligence approaches to understanding social intelligence is likely to advance both our understanding of ourselves, and the creation of socially intelligent machines which can interact naturally with people.

A particularly critical aspect of the challenge of building computational models of other minds—inferring emotional states—is taken up by Houlihan *et al*. [9] in their paper ‘Emotion prediction as inference over a generative theory of mind'. They describe a computational model of emotion prediction, the Inferred Appraisals model, that uses inverse planning to infer mental states, which can include individual objectives but also ‘social preferences' such as preference for equity or the desire to maintain a good reputation in the eyes of others. They show how it is possible to learn a mapping between these appraisals and 20 labels for emotions (including joy, relief, guilt and envy), so that the model can quantitatively match human predictions concerning these predictions in high-stakes game-like interactions. The model shows how social preferences turn out to be important in predicting almost every emotion; and also captures the flexibility of human emotion attribution. This work provides a starting point for computational models of social interaction, crucial both for understanding the nature of human social behaviour and for building socially sensitive artificial systems.

Continuing the theme of social interaction, in the final contribution to this issue, Chater [10] asks ‘How could we make a social robot?’ He argues that human intelligence is inherently social and that the spectacular achievements of our species arise through our ability to cooperate, collaborate and cumulatively creating languages, social norms, organizational and political structure, legal and financial systems, and the mathematics science and technology. A genuinely social robot therefore would be an artificial agent able to ‘join in' fluently with human projects and activities, learning, collaboration and contributing alongside us. Chater argues that the cognitive foundation of this process is a style of reasoning known as ‘virtual bargaining' according to which a pair of intelligent agents is able to coordinate their thoughts and actions by each asking not merely ‘what should *I* think or do?' but ‘what should *we* think or do?' Chater argues that answering this question successfully involves simulating what the agent will agree were they able to engage in prior communication—each party needs to successfully simulate the outcome of hypothetical bargaining process. Chater illustrates the approach by drawing on prior experimental work in which people are shown to be astonishingly successful, and highly flexible, in the use of novel and very restricted communicative signals. Here, the challenge of virtual bargaining is to agree what a novel signal would most naturally be interpreted to mean. He argues that this process of virtual bargaining underpins communication ‘in the moment', and that distinctive human ability to engage in virtual bargaining underpins the gradual creation of natural language and complex systems of societal conventions. Successive communicative and collaborative improvizations, each of which provides useful precedents for the next, explains the gradual emergence of increasingly systematic patterns in language and behaviour, through processes of spontaneous order. Thus, the complex machinery underpinning human society arises, to paraphrase the words of Scottish Enlightenment philosopher Adam Ferguson, through human action but not by human design. Chater suggests that by apparently skipping over the subtle process of virtual bargaining that underpins human communication, large language models (as discussed by Pavlick) may currently be missing out what may be a crucial step in understanding human social behaviour, and how it may be replicated in machines.

Overall, the contribution to this special issue on Cognitive artificial intelligence highlights convergent and overlapping research in both cognitive science and in artificial intelligence which is likely to be crucial to building both the next generation of increasingly human-like artificial systems and also providing a deeper understanding of the human mind.

## Data Availability

This article has no additional data.
